# Nutritional parameters affecting severity of pneumonia and length of hospital stay in patients with pneumococcal pneumonia: a retrospective cross-sectional study

**DOI:** 10.1186/s12890-015-0143-7

**Published:** 2015-11-25

**Authors:** Nobuhiro Akuzawa, Hiroshi Naito

**Affiliations:** Nutrition Support Team, Gunma Chuo Hospital, 1-7-13 Koun-cho, Maebashi, Gunma 371-0025 Japan

**Keywords:** CURB-65, Nutrition, Length of stay, Pneumococcal pneumonia, Pneumonia severity index

## Abstract

**Background:**

Pneumococcal pneumonia is the most common form of community-acquired pneumonia (CAP). Although a pneumococcal conjugate vaccine has contributed to a reduction in the incidence of pneumococcal pneumonia among older children and adults, no significant decrease in the incidence has been observed among persons aged ≥65 years. A low body mass index and hypoalbuminemia are common in Japanese patients with CAP, but the association of other nutritional parameters with the severity of pneumonia or length of hospital stay in patients with pneumococcal pneumonia is unclear.

**Methods:**

Fifty-seven previously healthy inpatients who presented with pneumococcal pneumonia were divided into two groups: those aged ≥65 years (*n* = 36) and those aged <65 years (*n* = 21). Patients’ characteristics (the Confusion, Urea, Respiratory rate, Blood pressure, age >65 years (CURB-65) score), the pneumonia severity index (PSI), and inflammatory and metabolic nutritional parameters were compared between the two groups.

**Results:**

The older group showed significantly lower serum albumin and cholinesterase (ChE) levels. Multivariate linear regression analysis revealed that the PSI was positively correlated with age in both groups. In the younger age group, both the CURB-65 score and PSI showed significant negative correlations with the serum ChE level, and there was a significant negative correlation between the length of stay and serum total cholesterol (T-cho) level. In the older group, the fasting period, lymphocyte count, and age showed significant positive correlations with the length of stay. There was a significant negative correlation between the length of stay and serum albumin level, but no correlation with the serum ChE or T-cho levels, in the older patients.

**Conclusions:**

Our findings suggest that in patients aged <65 years, age and serum ChE and T-cho levels were associated with both the severity of pneumococcal pneumonia and length of stay. In contrast, the length of stay in older patients was associated with multiple factors that differed from those in younger patients. These differences may reflect age-related immunosenescence in older patients and a greater effect of serum ChE and T-cho levels on immunity in younger patients.

## Background

*Streptococcus pneumoniae* (pneumococcus) is the most common cause of community-acquired pneumonia (CAP) [1]. The mortality rate of pneumococcal pneumonia is approximately 12 % and has not dramatically changed in the past 50 years despite medical advances [2]. A pneumococcal conjugate vaccine has contributed to a reduction in the incidence of disease among older children and adults [3–5]; however, no significant decrease in the incidence of invasive pneumococcal disease has been observed among persons aged >64 years [5]. Age of ≥65 years is also an independent predictive factor for in-hospital mortality of patients with CAP [6], and risk scores for patients with CAP, such as the Confusion, Urea, Respiratory rate, Blood pressure, age >65 years (CURB-65) score and pneumonia severity index (PSI), set a high value for age in patients with CAP [6, 7]. Moreover, the incidence of CAP tends to increase with age among people aged ≥65 years [8]. Age was reported to be significantly associated with in-hospital death only in patients with CAP aged >85 years with chronic obstructive disease, and a patient age of 65 to 84 years showed no association with in-hospital death [9]. This suggests that factors other than age itself, but that depend on age, are associated with CAP severity in patients ≥65 years old. A recent study reported a higher frequency of a low body mass index (BMI) (<18.0 kg/m^2^) and hypoalbuminemia (≤3.5 g/dL) in older Japanese patients with CAP than in control participants and found that hypoalbuminemia was associated with a significantly increased risk of pneumonia [10]. These finding suggest that malnutrition may be an important risk factor for CAP. Another study revealed that physical activity, nutritional status, and dehydration were significant prognostic factors in very old patients with pneumonia [11]. Moreover, in patients with CAP, a low serum albumin level (≤3.0 g/dL) and a low BMI during hospitalization were associated with death from pneumonia during a follow-up period after discharge [12]. However, these studies included patients with aspiration pneumonia or pneumonia resulting from bacteria other than *S. pneumonia* [8, 12]. The primary objectives of this study were to examine the differences in metabolic nutritional parameters between patients aged ≥65 and <65 years with pneumococcal pneumonia and to elucidate the association between metabolic nutritional parameters and the severity of pneumococcal pneumonia at admission and during hospitalization.

## Methods

### Study design and population

In total, 134 consecutive patients (52 male, 82 female) who were admitted to the Department of Internal Medicine in our hospital for the treatment of pneumococcal pneumonia from April 2009 to March 2014 were retrospectively identified in our departmental database. Diagnosis of pneumococcal pneumonia was confirmed by (i) detection of *S. pneumoniae* from a sputum culture, (ii) detection of *S. pneumonia* antigen in urine, or (iii) findings suggestive of bacterial pneumonia on imaging modalities such as X-ray and computed tomography.

Of these patients, 57 (21 male, 36 female) who had no remarkable medical history (including pneumococcus vaccination), were taking no medications, showed good activities of daily living, and had a full Functional Independence Measure (FIM) score were included in this study. None of the patients enrolled in this study needed mechanical ventilation support. All patients could be discharged from our hospital with a full FIM score and with no sequelae.

The study was conducted in accordance with the Declaration of Helsinki and approved by the Gunma Chuo Hospital Ethics Committee. Written informed consent for inclusion in the study was obtained from all enrolled patients.

### Clinical and laboratory findings

Patients were divided into two groups according to their age upon admission: those ≥65 years old (*n* = 36; age range, 65–90 years) and those <65 years old (*n* = 21; age range, 20–64 years). The mean age, sex ratio, BMI, CURB-65 score, and PSI were compared between these two groups. The CURB-65 is a scoring system that predicts mortality in patients with CAP [6]. The PSI is also a useful clinical tool for medical practitioners to calculate the probability of morbidity and mortality among patients with CAP [7]. The numbers of patients diagnosed with severe pneumonia (CURB-65 score of ≥4 and/or PSI of >130) were compared between the two groups. Inflammatory parameters (white blood cell count and serum C-reactive protein level) and metabolic nutritional parameters (lymphocyte count and serum levels of albumin [Alb], total cholesterol [T-cho], and cholinesterase [ChE]) [13, 14] were also compared between the two groups. Whole blood and plasma samples were obtained soon after admission.

### Antibiotic therapy and nutritional support

The patients were given antibiotic treatment in accordance with the 2005 guidelines for the management of CAP in adults provided by the Japanese Respiratory Society [15], the validity of which was proven in 2012 by a nationwide study in Japan [16]. The guideline committee recommended intravenous administration of high-dose penicillin, ceftriaxone, fourth-generation cephalosporins, carbapenems, or vancomycin for initial treatment of inpatients with pneumococcal pneumonia. In this study, the attending physicians chose a intravenous antibiotic, including high-dose aminobenzylpenicillin (6–8 g/day), ceftriaxone (2 g/day), or meropenem (2 g/day), which are frequently prescribed for primary treatment of CAP in our hospital. None of the enrolled patients were treated with oral or intravenous corticosteroids.

On the day of admission, all patients received peripheral parenteral nutrition (PPN) with intravenous infusion of both lactated Ringer’s solution (500 mL/day) and BFLUID (500 mL/day) (Otsuka Pharmaceutical Co., Ltd., Tokushima, Japan). BFLUID is an infusion preparation with an energy concentration of 4.2 kcal/mL, a pH of 6.7, and an osmotic pressure ratio of 3.0. It includes sugar (10.7 % w/v), amino acids, electrolytes, and vitamin B1, but no fat emulsion. From the second day of admission onward, the dose of intravenous nutrition infusion was readjusted to a maximum of 1000 mL/day in patients who were unable to eat enough food. The addition of BFLUID infusion to the usual lactated Ringer’s solution was determined by the attending physician. PPN with BFLUID was terminated when patients could eat more than half of their meals. Differences in the length of hospital stay, fasting period, duration of PPN, caloric intake by oral ingestion or PPN, types of antibiotics selected, and duration of antibiotic administration were compared between the two age groups. Correlations between length of stay and independent variables including age, inflammatory parameters, metabolic nutritional parameters, fasting period, oral or parenteral caloric intake, PPN duration, CURB-65 score, and PSI were investigated. Multivariate stepwise linear regression analysis was also performed to determine the factors associated with length of stay.

### Statistical analysis

Continuous data are presented as mean ± standard deviation or *n* (%). Comparisons of the admission data between the two age groups were performed using the unpaired *t* test for parametric data and the Mann–Whitney *U* test for nonparametric data. Nonparametric data, such as numbers of patients, were compared between the two groups using the χ^2^ test. Pearson’s correlation coefficient was used to analyze correlations between the CURB-65 score or PSI and independent variables, including age, BMI, and metabolic nutritional parameters (lymphocyte count and serum Alb, T-cho, and ChE levels). Similarly, Pearson’s correlation coefficient was used to analyze correlations between length of stay and independent variables including age, nutritional status parameters, fasting period, oral or parenteral caloric intake, PPN duration, CURB-65 score, and PSI. A *P*-value of <0.05 was considered to indicate a statistically significant association in the univariate analysis. Multivariate stepwise regression analysis was then performed to specify the independent variables showing a significant association with the CURB-65 score and PSI on admission as well as the length of stay in both groups. Basically, independent variables with a *P*-value of <0.1 in the univariate analysis were chosen for the multivariate analysis. Multicollinearity was assessed using the variance inflation factor. A variance inflation factor of >10 indicates serious multicollinearity, and a value of >4 may be a cause for concern. The variables that were found to be significantly or closely associated in the univariate analyses were included in the multivariate stepwise linear regression analyses, with *P* < 0.05 considered statistically significant. All analyses were performed using IBM SPSS Statistics for Windows, version 21.0 J (IBM Corp., Armonk, NY, USA). Datasets used for statistical analysis could not be published because Gunma Chuo Hospital Ethics Committee prohibits release of individual data to the public.

## Results

The characteristics of the patients in the two age groups are shown in Table 1. There were no significant differences in the sex ratio or BMI between the two groups. Interestingly, both groups showed a low BMI. In particular, the BMI in the ≥65-year-old group was nearly the lower limit of normal (18.5 kg/m^2^) [17]. The CURB-65 score was significantly higher in the older group than in the younger group (1.9 ± 0.9 vs 0.5 ± 0.9, respectively; *P* < 0.001), as was the PSI (105.6 ± 24.5 vs 28.2 ± 46.2, respectively; *P* < 0.001). The proportion of patients who presented with severe pneumonia was higher in the older group (25 % vs 5 %, respectively), but the difference between the two groups did not reach statistical significance (*P* = 0.052). Among the inflammatory and metabolic nutritional parameters, the older group had significantly lower levels of serum Alb (3.27 ± 0.59 vs 3.76 ± 0.64 g/dL, respectively; *P* = 0.005) and ChE (189.8 ± 54.3 vs 237.0 ± 78.1 U/L, respectively; *P* = 0.010).

**Table 1:** Comparison of patient characteristics and inflammatory and nutritional parameters between the ≥65- and <65-year-old groups

	≥65 years (*n* = 36)	<65 years (*n* = 21)	*P*-value
*Patients’ characteristics*			
Mean age (years)	80.1 ± 7.7	44.5 ± 13.9	<0.001*
Sex (male/female)	13/23	12/9	0.123
Body mass index (kg/m^2^)	18.7 ± 4.1	19.6 ± 2.1	0.284
CURB-65 score on admission	1.9 ± 0.9	0.5 ± 0.9	<0.001*
PSI on admission	105.6 ± 24.5	28.2 ± 46.2	<0.001*
*Inflammatory parameters*			
White blood cell count (/mm^3^)	12,750 ± 6490	13,300 ± 5460	0.744
C-reactive protein (mg/dL)	12.57 ± 11.97	9.60 ± 9.56	0.297
*Nutritional parameters*			
Lymphocyte count (/mm^3^)	1415.6 ± 1211.5	1664.0 ± 531.0	0.378
Serum albumin (g/dL)	3.27 ± 0.59	3.76 ± 0.64	0.005*
Total cholesterol (mg/dL)	167.5 ± 49.7	170.3 ± 43.5	0.833
Serum cholinesterase (IU/L)	189.8 ± 54.3	237.0 ± 78.1	0.010*

*CURB-65* confusion, urea, respiratory rate, blood pressure, age >65 years, *PSI* pneumonia severity index

*Statistically significant (*P* < 0.05)

### Correlation between CURB-65 score or PSI and independent variables on admission

The CURB-65 score on admission in the older group showed no significant correlations among the independent variables, including age, BMI, and metabolic nutritional parameters (Table 2A). The CURB-65 score in the younger group was significantly correlated with the age of the patients. The PSI on admission was significantly correlated with patient age in both groups (Table 2B).

**Table 2:** Correlation between CURB-65 score/PSI and independent variables including age, body mass index, and nutritional parameters

A. Univariate analysis of correlations between CURB-65 score and independent variables		
Independent variables	≥65 years (*n* = 36)	<65 years (*n* = 21)
Age	0.235 (0.167)	0.463 (0.034*)
Body mass index	−0.025 (0.883)	−0.210 (0.361)
Lymphocyte count	−0.164 (0.338)	−0.197 (0.392)
Serum albumin	−0.249 (0.142)	0.015 (0.948)
Total cholesterol	−0.248 (0.146)	−0.270 (0.237)
Serum cholinesterase	−0.122 (0.477)	−0.424 (0.056)
B. Univariate analysis of correlations between PSI and independent variables		
Independent variables	≥65 years (*n* = 36)	<65 years (*n* = 21)
Age	0.467 (0.004*)	0.640 (0.002*)
Body mass index	−0.084 (0.627)	−0.012 (0.958)
Lymphocyte count	−0.263 (0.122)	−0.145 (0.531)
Serum albumin	−0.156 (0.365)	−0.076 (0.744)
Total cholesterol	−0.287 (0.089)	−0.016 (0.946)
Serum cholinesterase	−0.148 (0.388)	−0.367 (0.102)

*P < 0.05. Data are presented as correlation coefficient (*P-*value)

Multivariate stepwise linear regression analysis was then carried out to determine the factors associated with the CURB-65 score and PSI in both groups. In the older group, a multivariate regression analysis of the CURB-65 score could not be performed because no independent variable had a *P-*value of <0.10 in the univariate analysis. The PSI in the older group showed a weak correlation with patient age (correlation coefficient [*r*] = 0.467, *P* = 0.004) (Table 3).

**Table 3:** Correlation between CURB-65 score/PSI and independent variables including age, body mass index, and nutritional parameters

Multivariate linear regression analysis					
1. Factors associated with PSI in the ≥65-year-old group (*r* = 0.467; *r* ^2^ = 0.219; adjusted *r* ^2^ = 0.196)					
Independent variable	B (95 % CI)	β	*t*	VIF	*P*
Age	1.477 (0.504–2.450)	0.467	3.084	1.000	0.004*
2. Factors associated with CURB-65 score in the <65-year-old group (*r* = 0.618; *r* ^2^ = 0.382; adjusted *r* ^2^ = 0.313)					
Independent variables	B (95 % CI)	β	*t*	VIF	*P*
Age	0.030 (0.004–0.056)	0.450	2.429	1.001	0.026*
Serum cholinesterase	−0.005 (−0.009 to −0.000)	−0.409	−2.208	1.001	0.040*
3. Factors associated with PSI in the <65-year-old group (*r* = 0.728; *r* ^2^ = 0.529; adjusted *r* ^2^ = 0.477)					
Independent variables	B (95 % CI)	β	*t*	VIF	*P*
Age	2.096 (0.962–3.229)	0.629	3.886	1.001	0.001*
Serum cholinesterase	−0.205 (−0.406 to −0.004)	−0.347	−2.142	1.001	0.046*

(*r* = 0.467; *r* ^2^ = 0.219; 2 adjusted *r* ^2^ = 0.196)

*r* multiple correlation coefficient, *r* ^2^ coefficient of determination, *B* nonstandardized regression coefficient, *CI* confidence interval; *β* standardized regression coefficient; *VIF* variance inflation factor.

*P < 0.05

Interestingly, multivariate regression analysis revealed that both the CURB-65 score and PSI in the younger group were correlated with the ChE level, although the absolute value of the standardized regression coefficient (β) of patient age was larger than that of the serum ChE level. Specifically, a weak but significant negative correlation was observed between the CURB-65 score and ChE level (β = −0.409, *P* = 0.040) and between the PSI and serum ChE level (β = −0.347, *P* = 0.046).

### Clinical course during hospitalization

All patients enrolled in this study were discharged from the hospital with a full FIM score, and no patient had delayed discharge because of social or health issues such as refusal to leave the hospital or the need for additional rehabilitation to recover activities of daily living. None of the patients was readmitted to our hospital within 1 year after discharge. However, the clinical courses during hospitalization differed between the two age groups. Specifically, the hospitalization period was significantly longer in the older than younger group (17.5 ± 12.9 vs 9.0 ± 3.4 days, respectively; *P* = 0.001) (Table 4). The older group also had a significantly longer fasting period (2.7 ± 7.2 vs 0.2 ± 0.8 days, respectively; *P* = 0.022), PPN implementation period (8.4 ± 11.4 vs 2.7 ± 2.6 days, respectively; *P* = 0.014), and antibiotic administration period (8.9 ± 2.6 vs 6.7 ± 2.9 days, respectively; *P* = 0.002). In addition, caloric intake by oral ingestion was significantly lower in the older group (1357.9 ± 533.1 vs 1652.4 ± 366.5 kcal/day, respectively; *P* = 0.03), while the PPN caloric intake was significantly higher (266.9 ± 49.4 vs 144.8 ± 153.6 kcal/day, respectively; *P* = 0.034). Regarding the antibiotics selected for treatment of pneumococcal pneumonia, the proportion of patients prescribed meropenem was significantly higher in the older group.

**Table 4:** Comparison of clinical course, caloric intake, and selected antibiotics during hospitalization

	≥65 years (*n* = 36)	<65 years (*n* = 21)	*P-*value
*Clinical course during hospitalization*			
Length of stay (days)	17.5 ± 12.9	9.0 ± 3.4	0.001*
Fasting period (days)	2.7 ± 7.2	0.2 ± 0.8	0.022*
Duration of PPN implementation (days)	8.4 ± 11.4	2.7 ± 2.6	0.014*
Duration of antibiotic administration (days)	8.9 ± 2.6	6.7 ± 2.9	0.002*
*Mean caloric intake*			
Oral ingestion (kcal/day)	1357.9 ± 533.1	1652.4 ± 366.5	0.030*
PPN (kcal/day)	266.9 ± 49.4	144.8 ± 153.6	0.034*
*Selected antibiotics*			
High-dose ABPC	18	13	0.384
Ceftriaxone	6	6	0.288
Meropenem	12	2	0.044*

*PPN* peripheral parenteral nutrition, *ABPC* aminobenzylpenicillin

*P < 0.05

### Correlations between variables and length of stay

Correlation analysis of factors associated with length of stay revealed interesting results (Table 5). In the younger group, the serum Alb (*r* = −0.542, *P* = 0.011), T-cho (*r* = −0.558, *P* = 0.009), and ChE levels (*r* = −0.493, *P* = 0.023) and the fasting period (*r* = 0.403, *P* = 0.023) were significantly correlated with the length of stay. In the older group, the serum Alb (*r* = −0.527, *P* = 0.001) and ChE levels (*r* = −0.516, *P* = 0.001) and the fasting period (*r* = 0.567, *P* < 0.001) showed significant correlations with the length of stay, but the serum T-cho level did not show a significant correlation. In addition, age (*r* = 0.479, *P* = 0.003), BMI (*r* = −0.488, *P* = 0.003), oral caloric intake (*r* = −0.445, *P* = 0.007), and PPN duration (*r* = 0.832, *P* < 0.001) showed significant correlations with the length of stay only in the older group. Regarding parameters associated with pneumonia severity, only the CURB-65 score in the younger group showed a significant correlation with length of stay (*r* = 0.438, *P* = 0.047).

**Table 5:** Correlations between length of stay and independent variables in univariate analysis

Independent variables	≥65 years (*n* = 36)	<65 years (*n* = 21)
Age	0.479 (0.003*)	−0.062 (0.788)
Body mass index	−0.488 (0.003*)	−0.145 (0.532)
*Nutritional parameters*		
Lymphocyte count	0.305 (0.071)	−0.163 (0.480)
Serum albumin	−0.527 (0.001*)	−0.542 (0.011*)
Total cholesterol	0.244 (0.152)	−0.558 (0.009*)
Serum cholinesterase	−0.516 (0.001*)	−0.493 (0.023*)
*Parameters related to nutrition support*		
Fasting period	0.567 (<0.001*)	0.403 (0.070)
Oral caloric intake	−0.445 (0.007*)	0.077 (0.740)
PPN caloric intake	0.163 (0.342)	0.237 (0.301)
PPN duration	0.832 (<0.001*)	0.115 (0.620)
*Parameters associated with pneumonia severity*		
CURB-65 score	0.295 (0.146)	0.438 (0.047*)
PSI	0.146 (0.396)	0.282 (0.215)

*P < 0.05. Data are presented as correlation coefficient (*P-*value)

Multivariate linear regression analysis in the older patients revealed that the fasting period (β = 0.399, *P* = 0.005), lymphocyte count (β = 0.315, *P* = 0.012), and age (β = 0.267, *P* = 0.044) had a significant positive correlation with the length of stay and that only the serum Alb level had a significant negative correlation (β = −0.292, *P* = 0.024) (Table 6). In younger patients, however, the serum T-cho level (β = −0.558, *P* = 0.009) was the only significant independent variable and showed a negative correlation with the length of stay.

**Table 6:** Multivariate stepwise linear regression analysis of factors associated with length of stay

1. Factors associated with length of stay in the ≥65-year-old group (*r* = 0.778; *r* ^2^ = 0.605; adjusted *r* ^2^ = 0.554)					
Independent variables	B (95 % CI)	β	*t*	VIF	*P*
Fasting period	0.718 (0.239–1.198)	0.399	3.055	1.340	0.005*
Lymphocyte count	0.003 (0.001–0.006)	0.315	2.680	1.086	0.012*
Serum albumin	−6.345 (−11.778 to −0.913)	−0.292	−2.382	1.176	0.024*
Age	0.446 (0.012–0.880)	0.267	2.098	1.086	0.044*
2. Factors associated with hospitalization period in the <65-year-old group (*r* = 0.558; *r* ^2^ = 0.311; adjusted *r* ^2^ = 0.275)					
Independent variable	B (95 % CI)	β	*t*	VIF	*P*
Serum total cholesterol	−0.043 (−0.074 to −0.012)	−0.558	3.055	1.000	0.009*

*P < 0.05

## Discussion

This study focused on the correlation between nutritional status and disease severity and between nutritional status and length of hospital stay in patients with pneumococcal pneumonia. Interestingly, the mean BMI of both age groups nearly reached the lower limit of normal (18.5 kg/m^2^). Past studies have shown that a lower BMI is associated with increased mortality among patients with CAP [18, 19] and that a higher BMI is associated with reduced mortality at 30 days after admission in patients with pneumococcal or *Haemophilus* CAP [20]. In the present study, however, BMI showed no significant correlations with pneumonia severity on admission or length of stay in the multivariate regression analysis in either age group. The most probable reason for this is selection bias resulting from the small number of enrolled patients in this study. Most of the patients had a low BMI, leading to the inability to show a significant association between BMI and pneumonia severity or length of stay.

It is understandable that both the CURB-65 score and PSI on admission were higher in the older than younger group because patient age is a determinant of both scores [6, 7]. The CURB-65 score is commonly used to discriminate severely ill patients, while the PSI has been well validated for the assessment of patients with a low mortality risk [21]. Interestingly, multivariate regression analysis showed significantly negative correlations between the serum ChE level and the pneumonia severity score, including both the CURB-65 score and PSI, only in the younger group. To the best of our knowledge, an association between the serum ChE level and severity of CAP has not been reported previously. These findings suggest that the serum ChE level may indirectly affect the immune response of patients with CAP. Acetylcholine was recently reported to have immunosuppressive effects on macrophages or other immune cells containing the α7 nicotinic acetylcholine receptor by suppressing inflammatory cytokine production [22]. This regulatory pathway mainly involves a link between neurotransmitters in blood and macrophages [22]. Therefore, low serum ChE levels, including both acetylcholinesterase and butyrylcholinesterase, may enhance immunosuppression by accumulation of unhydrolyzed acetylcholine, leading to activation of the cholinergic anti-inflammatory pathway and resultant aggravation of pneumococcal pneumonia [22, 23]. Actually, the use of ChE inhibitors in hospitalized patients with dementia has been suggested to increase the risk of respiratory disease, including pneumonia [24]. However, it should again be noted that there was a significant correlation between patient age and the CURB-65 score/PSI in both age groups in the present study. Advancing age is closely related to weakened integrity of physical barriers as well as low immune responsiveness against invading pathogens because of respiratory tract immunosenescence [25]. In the multivariate analysis, the absence of a significant correlation between independent variables and the PSI (other than age) in the older group may suggest more pronounced effects of immunosenescence on pneumonia severity in older patients with CAP.

Regarding length of stay, only the serum T-cho level showed a significant negative correlation in the younger group in the multivariate regression analysis. Past studies have shown that a lower serum T-cho level is associated with a risk of CAP in young patients [26] and that a low serum high-density lipoprotein cholesterol level may be a poor prognostic factor in patients with severe CAP [27]. Recent studies have revealed that alveolar epithelial type II cells and alveolar macrophages receive cholesterol from circulating high-density and low-density lipoprotein [28]. Alveolar epithelial type II cells also play a pivotal role in alveolar injury repair and homeostatic maintenance [29]. Accordingly, a lower T-cho level may be associated with dysfunction of alveolar epithelial type II cells and alveolar macrophages, slowing the recovery of pneumococcal pneumonia. On the other hand, in the older group, the age, fasting period, and lymphocyte count showed a significant positive correlation with the length of stay, while the serum Alb level was the only independent variable showing a significant negative correlation in the multivariate regression analysis. These results revealed different independent factors associated with length of stay in the two age groups, suggesting that multiple factors related to immunosenescence may affect length of stay in the elderly [25]. Actually, older patients with CAP showed a lower systemic inflammatory response than did younger patients with CAP despite equivalent serum concentrations of proinflammatory cytokines [30]. The number and functions of T lymphocytes also decrease with age, and hypoalbuminemic malnutrition can depress the activities of T lymphocytes [31, 32]. Moreover, fasting can decrease the activities of both mucosal T and B cells in terms of their ability to maintain pulmonary lymphocyte populations and mucosal IgA secretion in the respiratory tract and intestinal mucosa [33, 34]; thus, immune dysfunction resulting from immunosenescence, hypoalbuminemia, or fasting may be associated with a prolonged length of stay in older patients. However, the reason why the lymphocyte count was positively correlated with the length of stay only in the older group in the present study despite the fact that there were no significant differences in the lymphocyte count between the older and younger groups remains unclear. Therefore, further research is required to clarify the functional alterations of lymphocytes in older patients with pneumococcal pneumonia.

### Limitations

This study is limited by the relatively small number of patients from a single institution, possible selection bias, and the retrospective cross-sectional design. We mainly used univariate and multivariate linear regression analyses to investigate the association between independent variables and the severity of pneumonia or length of stay. Therefore, the relationship between these variables is unclear. Similarly, the clinical effects of antibiotics on length of stay are unclear. In addition, the associations of serum low-density lipoprotein, high-density lipoprotein, lymphocyte subsets, and objective nutritional parameters with body composition such as lean body mass were not measured. Prospective cohort studies are needed to further explore these interactions.

## Conclusions

The severity of pneumococcal pneumonia was most strongly correlated with patient age in both the ≥65- and <65-year-old groups. Notably, in the younger group, the serum ChE level was negatively correlated with the severity of pneumonia and the serum T-cho level was the only independent variable that was negatively correlated with the length of stay. In the older group, the fasting period, lymphocyte count, and age were positively correlated with the length of stay, while the serum Alb level was negatively correlated. These differences may reflect age-related immunosenescence in older patients and the marked effects of the serum ChE and T-cho levels on immunity in younger patients.

## Abbreviations

Alb: albumin
BMI: body mass index
CAP: community-acquired pneumonia
ChE: cholinesterase
CURB-65: Confusion, Urea, Respiratory rate, Blood pressure, age >65 years
FIM: Functional Independence Measure
PPN: peripheral parenteral nutrition
PSI: pneumonia severity index
T-cho: total cholesterol
